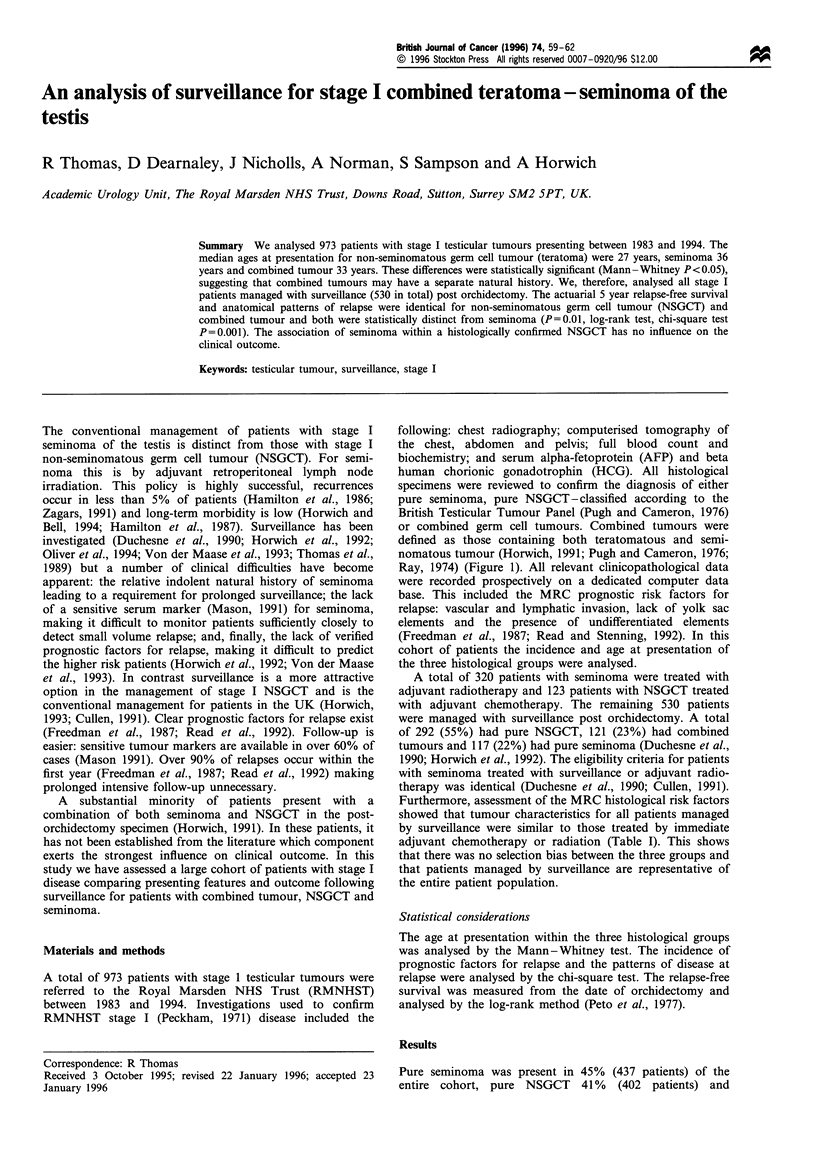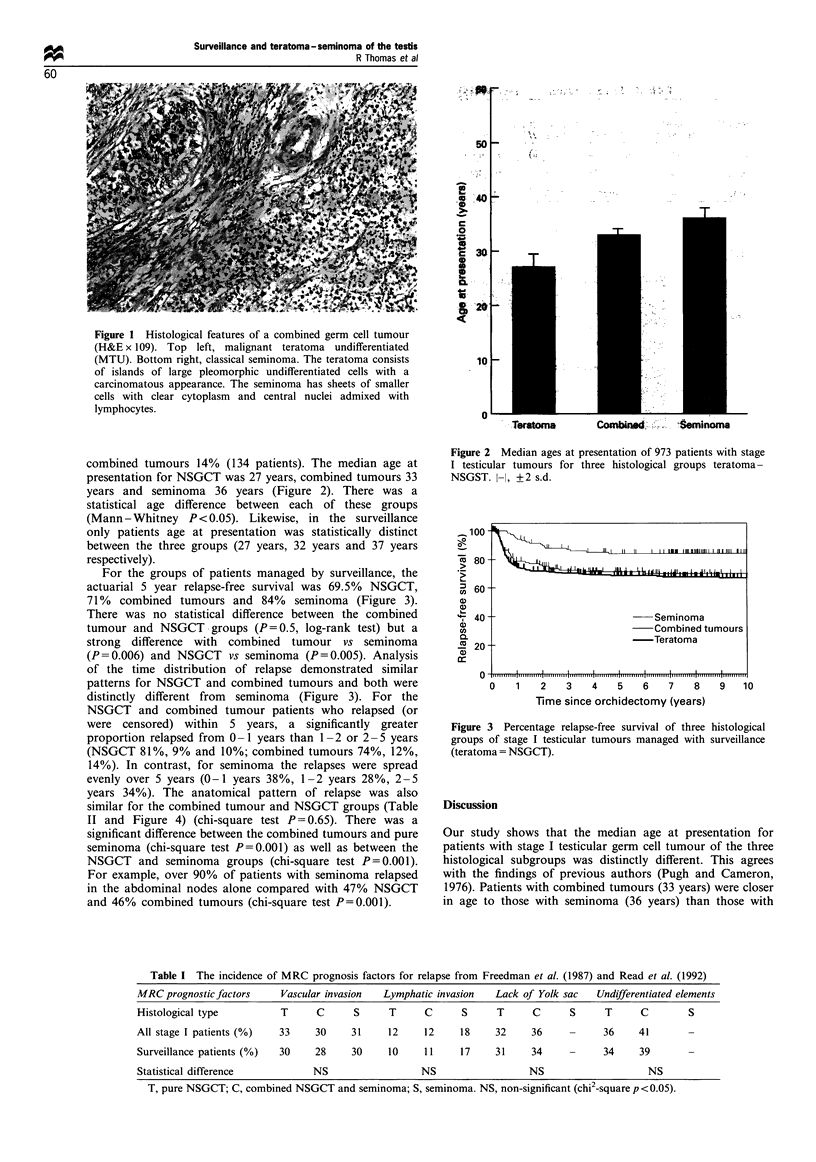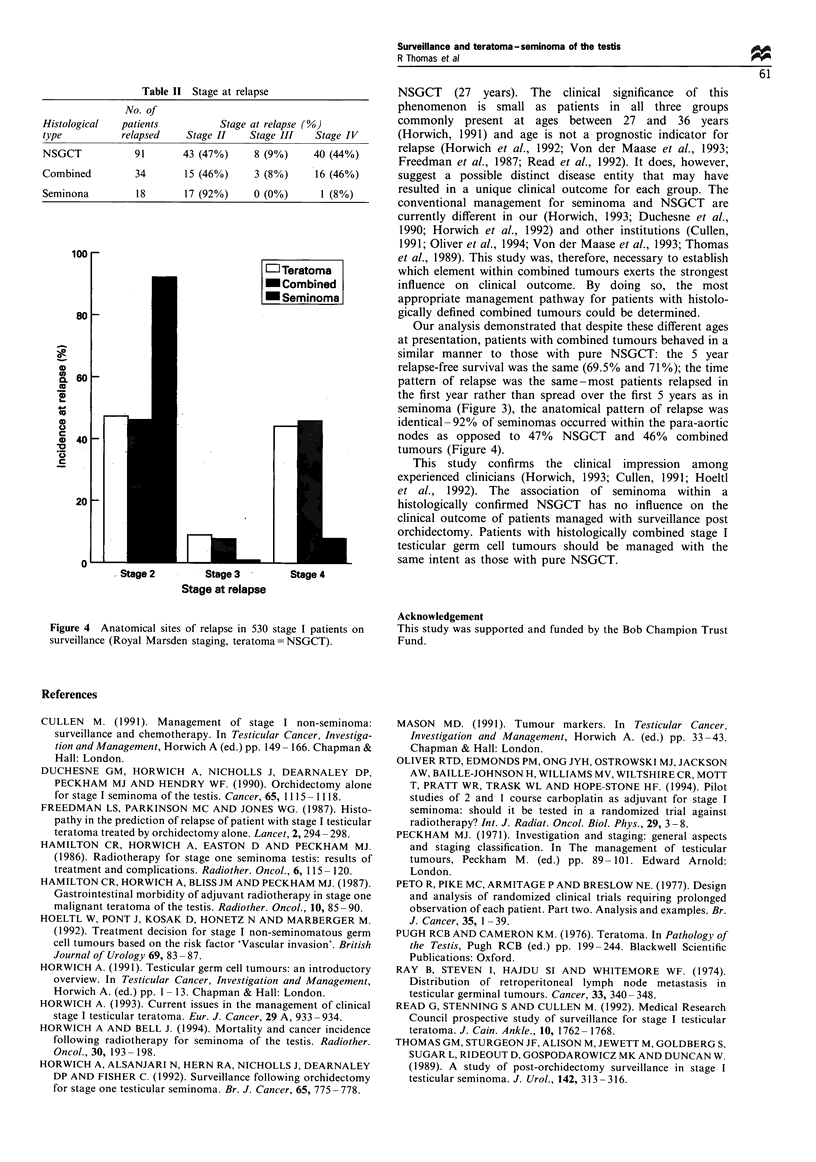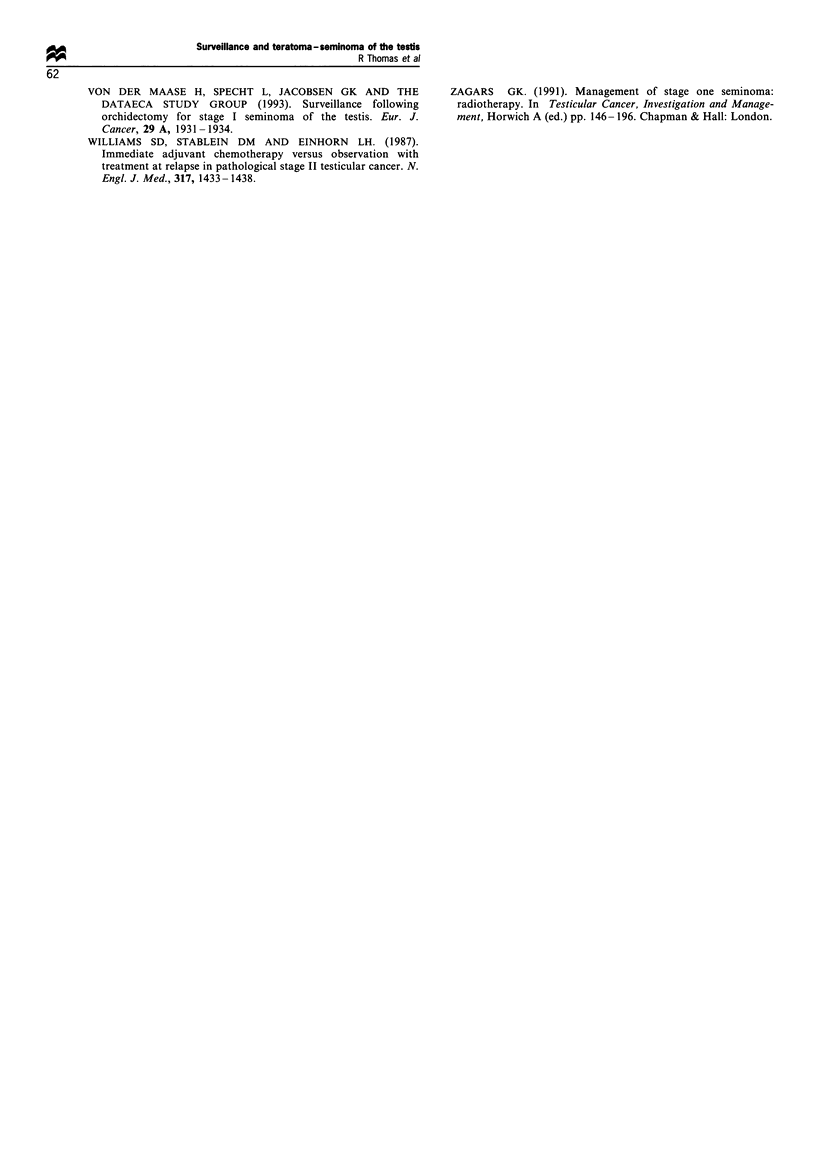# An analysis of surveillance for stage I combined teratoma--seminoma of the testis.

**DOI:** 10.1038/bjc.1996.315

**Published:** 1996-07

**Authors:** R. Thomas, D. Dearnaley, J. Nicholls, A. Norman, S. Sampson, A. Horwich

**Affiliations:** Academic Urology Unit, Royal Marsden NHS Trust, Sutton, Surrey, UK.

## Abstract

**Images:**


British Journal of Cancer (1996) 74, 59-62
© 1996 Stockton Press All rights reserved 0007-0920/96 $12.00

### An analysis of surveillance for stage I combined teratoma – seminoma of the testis

R Thomas, D Dearnaley, J Nicholls, A Norman, S Sampson and A Horwich

*Academic Urology Unit, The Royal Marsden NHS Trust, Downs Road, Sutton, Surrey SM2 5PT, UK.*

**Summary** We analysed 973 patients with stage I testicular tumours presenting between 1983 and 1994. The median ages at presentation for non-seminomatous germ cell tumour (teratoma) were 27 years, seminoma 36 years and combined tumour 33 years. These differences were statistically significant (Mann–Whitney $P<0.05$), suggesting that combined tumours may have a separate natural history. We, therefore, analysed all stage I patients managed with surveillance (530 in total) post orchidectomy. The actuarial 5 year relapse-free survival and anatomical patterns of relapse were identical for non-seminomatous germ cell tumour (NSGCT) and combined tumour and both were statistically distinct from seminoma ($P=0.01$, log-rank test, chi-square test $P=0.001$). The association of seminoma within a histologically confirmed NSGCT has no influence on the clinical outcome.

**Keywords:** testicular tumour, surveillance, stage I

The conventional management of patients with stage I seminoma of the testis is distinct from those with stage I non-seminomatous germ cell tumour (NSGCT). For seminoma this is by adjuvant retroperitoneal lymph node irradiation. This policy is highly successful, recurrences occur in less than 5% of patients (Hamilton *et al.*, 1986; Zagars, 1991) and long-term morbidity is low (Horwich and Bell, 1994; Hamilton *et al.*, 1987). Surveillance has been investigated (Duchesne *et al.*, 1990; Horwich *et al.*, 1992; Oliver *et al.*, 1994; Von der Maase *et al.*, 1993; Thomas *et al.*, 1989) but a number of clinical difficulties have become apparent: the relative indolent natural history of seminoma leading to a requirement for prolonged surveillance; the lack of a sensitive serum marker (Mason, 1991) for seminoma, making it difficult to monitor patients sufficiently closely to detect small volume relapse; and, finally, the lack of verified prognostic factors for relapse, making it difficult to predict the higher risk patients (Horwich *et al.*, 1992; Von der Maase *et al.*, 1993). In contrast surveillance is a more attractive option in the management of stage I NSGCT and is the conventional management for patients in the UK (Horwich, 1993; Cullen, 1991). Clear prognostic factors for relapse exist (Freedman *et al.*, 1987; Read *et al.*, 1992). Follow-up is easier: sensitive tumour markers are available in over 60% of cases (Mason 1991). Over 90% of relapses occur within the first year (Freedman *et al.*, 1987; Read *et al.*, 1992) making prolonged intensive follow-up unnecessary.

A substantial minority of patients present with a combination of both seminoma and NSGCT in the post-orchidectomy specimen (Horwich, 1991). In these patients, it has not been established from the literature which component exerts the strongest influence on clinical outcome. In this study we have assessed a large cohort of patients with stage I disease comparing presenting features and outcome following surveillance for patients with combined tumour, NSGCT and seminoma.

### Materials and methods

A total of 973 patients with stage 1 testicular tumours were referred to the Royal Marsden NHS Trust (RMNHST) between 1983 and 1994. Investigations used to confirm RMNHST stage I (Peckham, 1971) disease included the following: chest radiography; computerised tomography of the chest, abdomen and pelvis; full blood count and biochemistry; and serum alpha-fetoprotein (AFP) and beta human chorionic gonadotrophin (HCG). All histological specimens were reviewed to confirm the diagnosis of either pure seminoma, pure NSGCT–classified according to the British Testicular Tumour Panel (Pugh and Cameron, 1976) or combined germ cell tumours. Combined tumours were defined as those containing both teratomatous and seminomatous tumour (Horwich, 1991; Pugh and Cameron, 1976; Ray, 1974) (Figure 1). All relevant clinicopathological data were recorded prospectively on a dedicated computer data base. This included the MRC prognostic risk factors for relapse: vascular and lymphatic invasion, lack of yolk sac elements and the presence of undifferentiated elements (Freedman *et al.*, 1987; Read and Stenning, 1992). In this cohort of patients the incidence and age at presentation of the three histological groups were analysed.

A total of 320 patients with seminoma were treated with adjuvant radiotherapy and 123 patients with NSGCT treated with adjuvant chemotherapy. The remaining 530 patients were managed with surveillance post orchidectomy. A total of 292 (55%) had pure NSGCT, 121 (23%) had combined tumours and 117 (22%) had pure seminoma (Duchesne *et al.*, 1990; Horwich *et al.*, 1992). The eligibility criteria for patients with seminoma treated with surveillance or adjuvant radiotherapy was identical (Duchesne *et al.*, 1990; Cullen, 1991). Furthermore, assessment of the MRC histological risk factors showed that tumour characteristics for all patients managed by surveillance were similar to those treated by immediate adjuvant chemotherapy or radiation (Table I). This shows that there was no selection bias between the three groups and that patients managed by surveillance are representative of the entire patient population.

### *Statistical considerations*

The age at presentation within the three histological groups was analysed by the Mann–Whitney test. The incidence of prognostic factors for relapse and the patterns of disease at relapse were analysed by the chi-square test. The relapse-free survival was measured from the date of orchidectomy and analysed by the log-rank method (Peto *et al.*, 1977).

### Results

Pure seminoma was present in 45% (437 patients) of the entire cohort, pure NSGCT 41% (402 patients) and

Correspondence: R Thomas
Received 3 October 1995; revised 22 January 1996; accepted 23 January 1996

**Figure 1** Histological features of a combined germ cell tumour (H&E × 109). Top left, malignant teratoma undifferentiated (MTU). Bottom right, classical seminoma. The teratoma consists of islands of large pleomorphic undifferentiated cells with a carcinomatous appearance. The seminoma has sheets of smaller cells with clear cytoplasm and central nuclei admixed with lymphocytes.

**Figure 2** Median ages at presentation of 973 patients with stage I testicular tumours for three histological groups teratoma-NSGST. |-|, ±2 s.d.

combined tumours 14% (134 patients). The median age at presentation for NSGCT was 27 years, combined tumours 33 years and seminoma 36 years (Figure 2). There was a statistical age difference between each of these groups (Mann-Whitney $P<0.05$). Likewise, in the surveillance only patients age at presentation was statistically distinct between the three groups (27 years, 32 years and 37 years respectively).

For the groups of patients managed by surveillance, the actuarial 5 year relapse-free survival was 69.5% NSGCT, 71% combined tumours and 84% seminoma (Figure 3). There was no statistical difference between the combined tumour and NSGCT groups ($P=0.5$, log-rank test) but a strong difference with combined tumour *vs* seminoma ($P=0.006$) and NSGCT *vs* seminoma ($P=0.005$). Analysis of the time distribution of relapse demonstrated similar patterns for NSGCT and combined tumours and both were distinctly different from seminoma (Figure 3). For the NSGCT and combined tumour patients who relapsed (or were censored) within 5 years, a significantly greater proportion relapsed from 0-1 years than 1-2 or 2-5 years (NSGCT 81%, 9% and 10%; combined tumours 74%, 12%, 14%). In contrast, for seminoma the relapses were spread evenly over 5 years (0-1 years 38%, 1-2 years 28%, 2-5 years 34%). The anatomical pattern of relapse was also similar for the combined tumour and NSGCT groups (Table II and Figure 4) (chi-square test $P=0.65$). There was a significant difference between the combined tumours and pure seminoma (chi-square test $P=0.001$) as well as between the NSGCT and seminoma groups (chi-square test $P=0.001$). For example, over 90% of patients with seminoma relapsed in the abdominal nodes alone compared with 47% NSGCT and 46% combined tumours (chi-square test $P=0.001$).

**Figure 3** Percentage relapse-free survival of three histological groups of stage I testicular tumours managed with surveillance (teratoma = NSGCT).

### Discussion

Our study shows that the median age at presentation for patients with stage I testicular germ cell tumour of the three histological subgroups was distinctly different. This agrees with the findings of previous authors (Pugh and Cameron, 1976). Patients with combined tumours (33 years) were closer in age to those with seminoma (36 years) than those with

**Table I** The incidence of MRC prognosis factors for relapse from Freedman *et al.* (1987) and Read *et al.* (1992)

| *MRC prognostic factors* | *Vascular invasion* | | | *Lymphatic invasion* | | | *Lack of Yolk sac* | | | *Undifferentiated elements* | | |
|---|---|---|---|---|---|---|---|---|---|---|---|---|
| Histological type | T | C | S | T | C | S | T | C | S | T | C | S |
| All stage I patients (%) | 33 | 30 | 31 | 12 | 12 | 18 | 32 | 36 | – | 36 | 41 | – |
| Surveillance patients (%) | 30 | 28 | 30 | 10 | 11 | 17 | 31 | 34 | – | 34 | 39 | – |
| Statistical difference | | NS | | | NS | | | NS | | | NS | |

T, pure NSGCT; C, combined NSGCT and seminoma; S, seminoma. NS, non-significant (chi$^2$-square $p<0.05$).

**Table II** Stage at relapse

| *Histological type* | *No. of patients relapsed* | *Stage at relapse (%)* *Stage II* | *Stage III* | *Stage IV* |
|---|---|---|---|---|
| NSGCT | 91 | 43 (47%) | 8 (9%) | 40 (44%) |
| Combined | 34 | 15 (46%) | 3 (8%) | 16 (46%) |
| Seminona | 18 | 17 (92%) | 0 (0%) | 1 (8%) |

**Figure 4** Anatomical sites of relapse in 530 stage I patients on surveillance (Royal Marsden staging, teratoma = NSGCT).

NSGCT (27 years). The clinical significance of this phenomenon is small as patients in all three groups commonly present at ages between 27 and 36 years (Horwich, 1991) and age is not a prognostic indicator for relapse (Horwich *et al.*, 1992; Von der Maase *et al.*, 1993; Freedman *et al.*, 1987; Read *et al.*, 1992). It does, however, suggest a possible distinct disease entity that may have resulted in a unique clinical outcome for each group. The conventional management for seminoma and NSGCT are currently different in our (Horwich, 1993; Duchesne *et al.*, 1990; Horwich *et al.*, 1992) and other institutions (Cullen, 1991; Oliver *et al.*, 1994; Von der Maase *et al.*, 1993; Thomas *et al.*, 1989). This study was, therefore, necessary to establish which element within combined tumours exerts the strongest influence on clinical outcome. By doing so, the most appropriate management pathway for patients with histologically defined combined tumours could be determined.

Our analysis demonstrated that despite these different ages at presentation, patients with combined tumours behaved in a similar manner to those with pure NSGCT: the 5 year relapse-free survival was the same (69.5% and 71%); the time pattern of relapse was the same – most patients relapsed in the first year rather than spread over the first 5 years as in seminoma (Figure 3), the anatomical pattern of relapse was identical – 92% of seminomas occurred within the para-aortic nodes as opposed to 47% NSGCT and 46% combined tumours (Figure 4).

This study confirms the clinical impression among experienced clinicians (Horwich, 1993; Cullen, 1991; Hoeltl *et al.*, 1992). The association of seminoma within a histologically confirmed NSGCT has no influence on the clinical outcome of patients managed with surveillance post orchidectomy. Patients with histologically combined stage I testicular germ cell tumours should be managed with the same intent as those with pure NSGCT.

**Acknowledgement**

This study was supported and funded by the Bob Champion Trust Fund.

### References

CULLEN M. (1991). Management of stage I non-seminoma: surveillance and chemotherapy. In *Testicular Cancer, Investigation and Management*, Horwich A (ed.) pp. 149–166. Chapman & Hall: London.

DUCHESNE GM, HORWICH A, NICHOLLS J, DEARNALEY DP, PECKHAM MJ AND HENDRY WF. (1990). Orchidectomy alone for stage I seminoma of the testis. *Cancer*, **65**, 1115–1118.

FREEDMAN LS, PARKINSON MC AND JONES WG. (1987). Histopathy in the prediction of relapse of patient with stage I testicular teratoma treated by orchidectomy alone. *Lancet*, **2**, 294–298.

HAMILTON CR, HORWICH A, EASTON D AND PECKHAM MJ. (1986). Radiotherapy for stage one seminoma testis: results of treatment and complications. *Radiother. Oncol.*, **6**, 115–120.

HAMILTON CR, HORWICH A, BLISS JM AND PECKHAM MJ. (1987). Gastrointestinal morbidity of adjuvant radiotherapy in stage one malignant teratoma of the testis. *Radiother. Oncol.*, **10**, 85–90.

HOELTL W, PONT J, KOSAK D, HONETZ N AND MARBERGER M. (1992). Treatment decision for stage I non-seminomatous germ cell tumours based on the risk factor 'Vascular invasion'. *British Journal of Urology* **69**, 83–87.

HORWICH A. (1991). Testicular germ cell tumours: an introductory overview. In *Testicular Cancer, Investigation and Management*, Horwich A. (ed.) pp. 1–13. Chapman & Hall: London.

HORWICH A. (1993). Current issues in the management of clinical stage I testicular teratoma. *Eur. J. Cancer*, **29** A, 933–934.

HORWICH A AND BELL J. (1994). Mortality and cancer incidence following radiotherapy for seminoma of the testis. *Radiother. Oncol.*, **30**, 193–198.

HORWICH A, ALSANJARI N, HERN RA, NICHOLLS J, DEARNALEY DP AND FISHER C. (1992). Surveillance following orchidectomy for stage one testicular seminoma. *Br. J. Cancer*, **65**, 775–778.

MASON MD. (1991). Tumour markers. In *Testicular Cancer, Investigation and Management*, Horwich A. (ed.) pp. 33–43. Chapman & Hall: London.

OLIVER RTD, EDMONDS PM, ONG JYH, OSTROWSKI MJ, JACKSON AW, BAILLE-JOHNSON H, WILLIAMS MV, WILTSHIRE CR, MOTT T, PRATT WR, TRASK WL AND HOPE-STONE HF. (1994). Pilot studies of 2 and 1 course carboplatin as adjuvant for stage I seminoma: should it be tested in a randomized trial against radiotherapy? *Int. J. Radiat. Oncol. Biol. Phys.*, **29**, 3–8.

PECKHAM MJ. (1971). Investigation and staging: general aspects and staging classification. In The management of testicular tumours, Peckham M. (ed.) pp. 89–101. Edward Arnold: London.

PETO R, PIKE MC, ARMITAGE P AND BRESLOW NE. (1977). Design and analysis of randomized clinical trials requiring prolonged observation of each patient. Part two. Analysis and examples. *Br. J. Cancer*, **35**, 1–39.

PUGH RCB AND CAMERON KM. (1976). Teratoma. In *Pathology of the Testis*, Pugh RCB (ed.) pp. 199–244. Blackwell Scientific Publications: Oxford.

RAY B, STEVEN I, HAJDU SI AND WHITEMORE WF. (1974). Distribution of retroperitoneal lymph node metastasis in testicular germinal tumours. *Cancer*, **33**, 340–348.

READ G, STENNING S AND CULLEN M. (1992). Medical Research Council prospective study of surveillance for stage I testicular teratoma. *J. Cain. Ankle.*, **10**, 1762–1768.

THOMAS GM, STURGEON JF, ALISON M, JEWETT M, GOLDBERG S, SUGAR L, RIDEOUT D, GOSPODAROWICZ MK AND DUNCAN W. (1989). A study of post-orchidectomy surveillance in stage I testicular seminoma. *J. Urol.*, **142**, 313–316.

VON DER MAASE H, SPECHT L, JACOBSEN GK AND THE DATAECA STUDY GROUP (1993). Surveillance following orchidectomy for stage I seminoma of the testis. *Eur. J. Cancer*, **29 A,** 1931-1934.

WILLIAMS SD, STABLEIN DM AND EINHORN LH. (1987). Immediate adjuvant chemotherapy versus observation with treatment at relapse in pathological stage II testicular cancer. *N. Engl. J. Med.*, **317,** 1433-1438.

ZAGARS GK. (1991). Management of stage one seminoma: radiotherapy. In *Testicular Cancer, Investigation and Management,* Horwich A (ed.) pp. 146-196. Chapman & Hall: London.